# Comparing various AI approaches to traditional quantitative assessment of the myocardial perfusion in [^82^Rb] PET for MACE prediction

**DOI:** 10.1038/s41598-024-60095-6

**Published:** 2024-04-26

**Authors:** Sacha Bors, Daniel Abler, Matthieu Dietz, Vincent Andrearczyk, Julien Fageot, Marie Nicod-Lalonde, Niklaus Schaefer, Robert DeKemp, Christel H. Kamani, John O. Prior, Adrien Depeursinge

**Affiliations:** 1 Nuclear Medicine and Molecular Imaging Department, Lausanne University Hospital, Lausanne, Switzerland; 2 Department of Oncology, Lausanne University Hospital, Lausanne, Switzerland; 3 Institute of Informatics, School of Management, HES-SO Valais-Wallis University of Applied Sciences and Arts Western Switzerland, Sierre, Switzerland; 4 INSERM U1060, CarMeN laboratory, University of Lyon, Lyon, France; 5 AudioVisual Communications Laboratory (LCAV), EPFL, Lausanne, Switzerland; 6 University of Lausanne, Lausanne, Switzerland; 7 Division of Cardiology, University of Ottawa Heart Institute, Ottawa, ON Canada; 8 Department of Cardiology, Lausanne University Hospital, Lausanne, Switzerland

**Keywords:** Computational platforms and environments, Cardiology, Interventional cardiology, Computer science, Prognostic markers

## Abstract

Assessing the individual risk of Major Adverse Cardiac Events (MACE) is of major importance as cardiovascular diseases remain the leading cause of death worldwide. Quantitative Myocardial Perfusion Imaging (MPI) parameters such as stress Myocardial Blood Flow (sMBF) or Myocardial Flow Reserve (MFR) constitutes the gold standard for prognosis assessment. We propose a systematic investigation of the value of Artificial Intelligence (AI) to leverage [^82^Rb] Silicon PhotoMultiplier (SiPM) PET MPI for MACE prediction. We establish a general pipeline for AI model validation to assess and compare the performance of global (i.e. average of the entire MPI signal), regional (17 segments), radiomics and Convolutional Neural Network (CNN) models leveraging various MPI signals on a dataset of 234 patients. Results showed that all regional AI models significantly outperformed the global model ($p<0.001$), where the best AUC of 73.9% (CI 72.5–75.3) was obtained with a CNN model. A regional AI model based on MBF averages from 17 segments fed to a Logistic Regression (LR) constituted an excellent trade-off between model simplicity and performance, achieving an AUC of 73.4% (CI 72.3–74.7). A radiomics model based on intensity features revealed that the global average was the least important feature when compared to other aggregations of the MPI signal over the myocardium. We conclude that AI models can allow better personalized prognosis assessment for MACE.

## Introduction

Major Adverse Cardiac Events (MACE) commonly designate stroke, Myocardial Infarction (MI) or cardiac death. As of 2023, those events are the most common outcomes of cardiovascular diseases and remain among the leading causes of death across the world^1^. Personalized patient prognosis and the prediction of MACE is therefore a challenge of real interest. Well established risk factors include sedentarity, obesity, diabetes mellitus, arterial hypertension, dyslipidemia, smoking history, and family history of cardiovascular disease^2–4^. All those clinical features are not accurate enough when employed for patient personalized prediction, since a significant proportion of the population with cardiovascular diseases does not manifest any of those classical risk factors^5^.

Myocardial Perfusion Imaging (MPI) using Single-Photon Emission Computed Tomography (SPECT) is the most frequently performed nuclear cardiology procedure. It is a sensitive tool for the detection, the localization, and the risk stratification of ischemic heart disease, as well as the assessment of Left Ventricular (LV) function, and myocardial viability. Furthermore, MPI based on Magnetic Resonance Imaging (MRI) is used for its anatomic detail, tissue contrast, spatial and temporal resolution, as well as the lack of ionizing radiation^6^. As an alternative, quantitative MPI can be obtained using [^82^Rb] Positron Emission Tomography (PET) and is nowadays a functional and non-invasive method for assessing the risk of MACE, or to investigate physiological consequences on the organ after a cardiac event^7–12^. It is already known to bring a strong predictive value for MACE prediction^13^ and was proven cost effective^14^. This procedure quantifies the Myocardial Blood Flow (MBF) and the Myocardial Flow Reserve (MFR) in the LV using dynamic [^82^Rb] PET/CT acquisitions. MBF at stress (sMBF) and rest (rMBF) can be accurately quantified in ml/min/g^15^. The MFR constitutes the ratio of MBF during maximal coronary vasodilatation to resting MBF and is therefore impacted by both rest and stress flow. Thus, it represents the relative reserve of the coronary circulation. Both MBF and MFR are then mapped onto a Polar Map (PM) for visualization. PM visualization yields a controlled positioning and parcellation of all subregions of the LV across patients and acquisitions, based on the 17-segment model of the American Heart Association (AHA)^16^.

Various alterations of the MBF were identified to be associated with an increased risk of MACE. In particular, physiological expectations of global MBF impairment are related to multi-vessel epicardial disease or microcirculatory dysfunction. Global sMBF (i.e. average across the entire PM as a measure of the total perfusion of the LV) is known to be predictive for MACE^13^. More isolated alterations of the myocardial perfusion could be related to small defects caused by epicardial coronary artery disease^17^. To further investigate this hypothesis, Gould et al. introduced the Myocardial Flow Capacity (MFC) combining sMBF and MFR in a two dimensional representation^17^ for categorizing MACE risk.

Nevertheless, the wealth and complexity of the information contained in PMs can be difficult to fully leverage with the naked eye (e.g. micro lesions or subtle patterns) and are subject to inter- and intra- observer variability with semiquantitative visual assessment alone^18^. Better individualized risk estimates, with epidemiological effectiveness but also cost efficiency are needed. Artificial Intelligence (AI) has the potential to fully exploit the information provided by PET MPI and dramatically enhance the utility of this powerful modality. Machine Learning (ML)-based PM interpretation already surpassed the clinical interpretation of SPECT MPI for MACE prediction^19^. The incorporation of AI techniques to standardize and automate processing of PET MPI could further improve cardiovascular risk stratification^20,21^ to noninvasively support clinical decision for using coronary revascularization^9^ and guide the clinical management of patients with suspected coronary artery disease. This would provide an accurate and systematic assessment of tissue perfusion hemodynamics in a one-stop-shop.

In the specific context of MPI assessed via nuclear medicine and molecular imaging, the very first AI studies originated in the 90s. A large body of literature focused on SPECT due to its large usage and availability^22^. In 1995, Hamilton et al. used a three-layered Multi-Layer Perceptron (MLP) fed by vectorized 24 territorial values of PMs from [^201^TI] SPECT to classify normal and abnormal regions with an AUC (Area Under the receiver operating characteristic Curve) of 96%^23^. In 2020, Slomka et al. established the REgistry of Fast Myocardial Perfusion Imaging with NExt generation SPECT/CT (REFINE SPECT)^24^, an outstanding data resource with MACE as the primary endpoint. It includes more than 20,000 patients from nine centers with [^99m^Tc] SPECT along with extensive additional relevant parameters concerning patient data, ECG and treatment. Using the REFINE SPECT database, the prognostic performance of the semi-quantitative assessment of SPECT PMs via stress Total Perfusion Deficit (TPD)^25^ was found to be superior to visual assessment for predicting MACE^26^. Betancur et al. used LogitBoost^27^, a ML approach based on clinical and semi-quantitative imaging variables from [^99m^Tc] SPECT (e.g. TPD) to predict MACE (3-year risk), which outperformed visual and semi-quantitative assessments^28^.

Radiomics aims at extracting large collection of quantitative image measurements describing intensity, shape and texture of regions of interest. Its value was also investigated in the context of nuclear cadiology, with prediction of dilated cardiomyopathy in SPECT MPI^29^, contraction patterns in gated SPECT MPI^30^, normal/abnormal and low-risk/high-risk classification in SPECT^31^ as well as detection of diffusely impaired myocardial perfusion in [^13^N] ammonia PET MPI^32^. The stability of radiomics features across SPECT scanners was investigated in^33^.

Deep Learning (DL) is a subcategory of ML that can directly use images as input. It can deduce and extract optimal image features for the task at hand, obviating the need to handcraft specific features as it is the case for radiomics ML models for instance. [^99m^Tc] SPECT-based DL using a Convolutional Neural Network (CNN) was found to be marginally more predictive of obstructive Coronary Artery Disease (CAD) when compared to semi-quantitative assessment via stress TPD^34^. The accuracy of a CNN based on multiple channels input including sMBF, LV wall motion and wall thickening maps obtained from [^99m^Tc] SPECT/CT for the diagnosis of obstructive CAD was assessed by the REFINE SPECT study^35^. Apostolopoulos et al. used CNNs with [^99m^Tc] SPECT PMs to classify 216 patients with either flow-limiting- or no- CAD, which reach performance on par with physicians^36,37^. Spier et al. compared various DL approaches to classify between normal and abnormal [^99m^Tc] SPECT stress and rest PMs^38^. In particular, they compare MLPs, CNNs on flattened PMs (i.e. either reshaping the PM pixel organization to a square grid, or padding the corners with zeros) and Graph CNNs (GCNNs). A large performance gain is observed with GCNNs when compared to CNNs and MLPs, suggesting that adequate consideration of PM geometry is crucial.

First studies on the relevance of AI based on PET in coronary artery disease were recently published^39^. Juarez-Orozco et al. used LogitBoost ML with 4 demographic, 8 clinical, and 9 functional variables from [^13^N] ammonia PET/CT to predict myocardial ischemia and MACE^40^. Wang et al.^41^ compared the performance of a Support Vector Machine (SVM) ML model based on 6 MPI variables derived from both [^13^N] ammonia and [^18^F]FDG PET/CT to predict the presence of vascular stenosis in patients with suspected obstructive CAD and achieved an AUC of 68%. A multi-task ML based on [^13^N] ammonia PET/CT was developed by Yeung et al. to identify impaired MFR as well as cardiovascular risk factors^42^. Kwiecinski et al. used ML based on [^18^F] sodium fluoride PET and quantitative plaque analysis on CT angiography to predict the risk of MI^43^.

To date, the potential of AI methods to leverage the wealth of [^82^Rb] PET has been little explored. Wang et al. used a MLP to increase MPI quality^44^ and DL was used to improve motion correction in^45^. Singh et al.^46^ used DL based on multi-channel [^82^Rb] PET PMs (i.e. stress and rest MBF, MFR, and spill-over fraction computed using QPET^47^) augmented with sex, shape indices^48^ and LV end-systolic and diastolic volumes to predict all-cause mortality. They reported significantly improved mortality prediction when compared to established measures of ischemia.

This study aims to provide a systematic exploration of the prognostic value of [^82^Rb] PET-based MPI analysis using AI for MACE prediction. To this end, we systematically evaluate and compare standard AI approaches from simple Logistic Regression (LR) based on handcrafted features (e.g. global or regional intensity or radiomics with and without texture features) to CNNs, as well as their combined performance with clinical data. In particular, we address the following research questions when considering MACE prediction: (i) do regional AI models outperform global sMBF (i.e. the traditional quantitative assessment^13^)? (ii) can spatial pattern analysis (i.e. radiomics texture or CNN) improve intensity aggregation (i.e. radiomics first order statistics)? (iii) can we combine MPI and clinical data to improve MACE prediction?

## Methods

This section systematically details the different steps followed across the study, starting with the descriptions of study population, images acquisition protocols, and data format. We then present all AI models considered and the evaluation pipeline for the classification task of predicting MACE based on [^82^Rb] PET.

### Studied population

Participants with suspected myocardial ischemia were enrolled to undergo [^82^Rb] cardiac Silicon PhotoMultiplier (SiPM) PET/CT at the Lausanne University Hospital between June 2018 and June 2019^13^. All their cardiovascular risk factors and medication use were determined at time of PET imaging. The Local Ethics Committee, Commission cantonale d’Éthique de la Recherche sur l’être humain VauD (CER-VD) approved this study protocol ($\#PB\_2017{-}00634$), and all participants gave written informed consent prior to inclusion. All methods were performed in accordance with the relevant guidelines and regulations. The dataset used for this study consists of MBF measurements at rest and under stress for 234 patients, along with a set of 19 clinical features for each patient (see Supplementary Material A). Among this cohort, there were 187 patients with no observed MACE event in the follow-up days and 47 patients labeled as having a MACE event. The median number of days between PET imaging and first MACE or last news was 652 (interquartile range 559–751). The following events were considered as MACE: cardiac death, delayed revascularization (more than 6 months post-PET/CT), MI, hospitalization for congestive heart failure, or de novo stable angina. MI was defined by clinical presentation compatible with an ST-segment elevation MI or a non-ST-elevation MI, angiographic confirmation of coronary artery disease in the appropriate territory, and an elevated troponin level. Baseline clinical characteristic are detailed in Table 1. More details concerning data acquisition can be found in Dietz et al.^13^.

**Table 1:** Baseline clinical characteristics^13^.

	Overall population (*n* = 234)	MACE (*n* = 47)	No MACE (*n* = 187)	*p*-value
Age, years	72 [61–78]	73 [68–79]	71 [60–77.5]	0.105
Male sex	153 (65%)	36 (77%)	117 (63%)	0.081
Body mass index, kg/m^2^	31 [28–36]	32 [28.5–35.5]	31 [28–36]	0.78
Cardiovascular risk factors				
Hypertension	171 (73%)	38 (81%)	133 (71%)	0.18
Current or former smoker	106 (45%)	26 (55%)	80 (43%)	0.12
Dyslipidemia	159 (68%)	34 (72%)	125 (67%)	0.47
Diabetes	85 (36%)	20 (43%)	65 (35%)	0.32
Insulin-requiring diabetes	35 (15%)	10 (21%)	25 (13%)	0.14
Known CAD	126 (54%)	34 (72%)	92 (49%)	0.004
History of MI	100 (43%)	28 (60%)	72 (39%)	0.01
Medications				
Aspirin	135 (58%)	28 (60%)	107 (57%)	0.35
Beta-blockers	144 (62%)	33 (70%)	111 (59%)	0.15
ACE inhibitors/ARB	135 (58%)	32 (68%)	103 (55%)	0.15
Diuretics	82 (35%)	27 (57%)	55 (29%)	0.0002
Nitroglycerine therapy	24 (10%)	6 (13%)	18 (10%)	0.61
Lipid-lowering agent	154 (66%)	32 (68%)	122 (65%)	0.71

Reported values are either median [interquartile range] or *n* (%).

*ACE* Angiotensin-Converting Enzyme, *ARB* Angiotensin Receptor Blocker.

### Imaging protocol

All subjects underwent a rest and adenosine or regadenoson stress SiPM PET/CT scan using a single scanner (Biograph Vision 600, Siemens Medical Solutions, Knoxville, USA). They fasted for 6 h and avoided caffeine 24 h before the test. At rest, a 15–25 s i.v. infusion of 5 MBq/kg of [^82^Rb] was injected with an automatic infusion system (Ruby-Fill®generator and [^82^Rb] elution system [v3], Jubilant DraxImage, Kirkland, QC, Canada). 3D dynamic PET images were acquired starting with the infusion over 6 min 6 s ($12\times 8$, $5\times 12$, $1\times 30$, $1\times 60$, and $1\times 120$ s). For stress acquisitions, adenosine (140 mg/min/kg over 6 min) or regadenoson (400 μg over 10–20 s) were administered i.v., followed by [^82^Rb] infusion at 2 min after adenosine and directly after regadenoson administration. The stress protocol was then similar to the rest acquisition. An accompanying low-dose CT (100 keV, 16 mAs) transmission scan was acquired for attenuation correction. An ordered subset expectation maximization algorithm was used for image reconstruction (4 iterations, 5 subsets, 4.0 mm FWHM gaussian post-filter, $220\times 220$ pixel matrix size). Blood pressure, heart rate, and a 12-channel ElectroCardioGram (ECG) were collected during the image acquisitions. Imaging characteristics are presented in Table 2.

**Table 2:** Myocardial perfusion imaging characteristics^13^.

	Overall population (*n* = 234)	MACE (*n* = 47)	No MACE (*n* = 187)	*p*-value
PET pharmacological stress agent				
Adenosine	204 (87%)	40 (85%)	164 (88%)	0.56
Hemodynamics during PET/CT				
Rest-HR, bpm	70 [61-78]	71 [61-81.5]	69 [61-75.5]	0.12
Stress-HR, bpm	83 [74-95]	82 [75-99]	85 [74-94.5]	0.72
Rest-SBP, mmHg	136 ± 23	134 ± 24	136 ± 22	0.6
Stress-SBP, mmHg	120 [104-137]	118 [101-131]	120 [107.5-138]	0.07
Rest-DBP, mmHg	71 ± 12	69 ± 11	71 ± 12.5	0.3
Stress-DBP, mmHg	61 [54-70]	60 [52-63.5]	62 [55-71]	0.11
Rest-RPP > 8500 mmHg/min	153 (65%)	29 (62%)	124 (66%)	0.60
^82^Rb quantitative imaging				
Global rMBF, mL/min/g	0.82 [0.65-1.06]	0.72 [0.51-0.925]	0.75 [0.59-0.97]	0.34
Global sMBF, mL/min/g	1.96 [1.32-2.71]	1.5 [1.08-1.87]	2.16 [1.54-2.88]	$< 0.0001$
Global sMBF $< 1.94$ mL/min/g	113 (48%)	37 (79%)	76 (41%)	$< 0.0001$
Global MFR	2.39 [1.72-3.0]	1.75 [1.395-2.47]	2.49 [1.93-3.1]	$< 0.0001$
Global MFR $< 1.98$	78 (33%)	29 (62%)	49 (26%)	$< 0.0001$

Reported values are either median [interquartile range] or *n* (%).

*DBP* Diastolic Blood Pressure, *HR* Heart rate, *SBP* systolic blood pressure, *RPP* rate-pressure product (*RPP* = *HR* $\times$ *SBP*).

### Quantitative myocardial perfusion assessment and data format

Perfusion was quantitatively assessed via MBF in ml/g/min at rest and stress, using the FlowQuant v2.7 software (Ottawa, Ontario, Canada) based on a 1-tissue compartment model and flow-dependent extraction correction^49^.

We established the rate-pressure product adjusted rest MBF and the resultant MFR in consideration of variations in resting flow due to differing haemodynamic conditions^50^. This was achieved by multiplying the rest MBF by 8500 mmHg/min and then dividing it by the rate-pressure product (resting heart rate multiplied by resting systolic blood pressure). A dual spill-over correction^51^ as well as global partial-volume recovery correction and motion correction^52^ were systematically applied to reduce the potential spillover in image-derived blood activity curves. Semi-automated segmentation of the myocardium was performed using FlowQuant.

**Figure 1:**
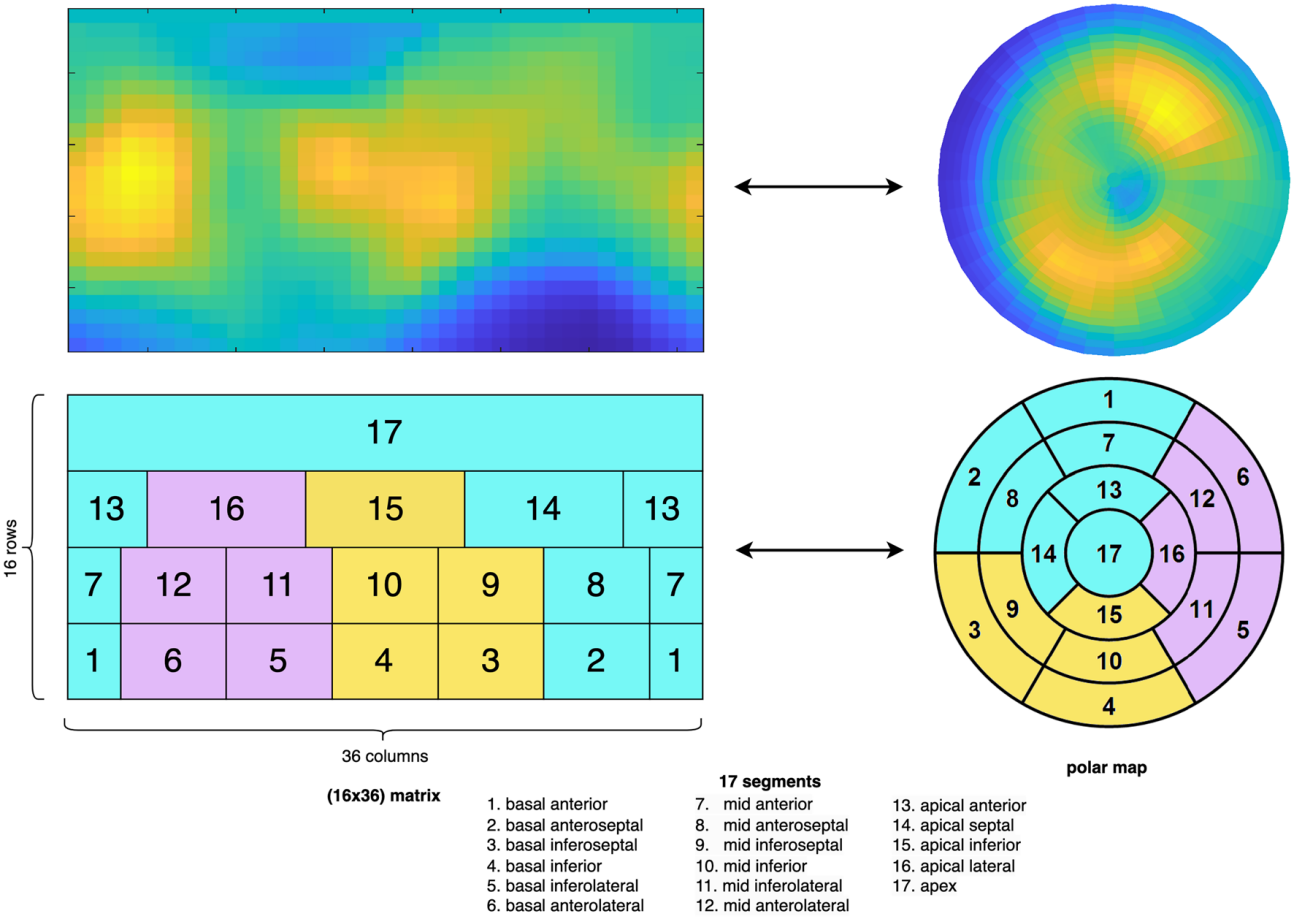
Mapping between $16\times 36$ measurement matrix (left) and Polar Map (PM) (right) representation. Top row: Example of MBF measurements in matrix and PM representation. Bottom row: PM representation of the myocardial perfusion with 17-segment AHA parcellation. 3-segment parcellation is depicted with yellow (inferior wall), purple (lateral wall) and cyan (anterior wall) colors. The mapping between the $16\times 36$ MBF matrix *S* (left) and the MBF PM (right) is depicted.

The MBF measurements are provided in the form of a polar-pixelized matrix stored in $24 \times 36$ matrices of nonnegative float numbers. The last 8 rows of those matrices were cut off to match with the set of values commonly used to produce the standard PMs representing the perfusion of the LV (see the mapping between the MBF matrix and the MBF PMs according to the 17-segment model in Fig. 1). A measurement matrix is denoted by $S = (S[m,n])_{1 \le m \le 16, 1 \le n \le 36} \in \mathbb {R}^{16 \times 36}$. Each value *S*[*m*, *n*] represents the perfusion signal at a given polar pixel, where *m* characterizes the radius to the pole and *n* the angular position of the polar pixel. Note that the matrix has a periodic structure in the sense that the columns 1 and 36 are spatial neighbors in the PM. MBF values were acquired under stress (sMBF) and at rest (rMBF), leading to two measurement matrices $S_{\text {sMBF}}$ and $S_{\text {rMBF}}$. For all image analysis approaches, we chose to work with the tabular format *S*[*m*, *n*] instead of the PM images. This allowed to avoid any kind of data transformation such as spatial resampling or intensity binning needed to construct the PM image, as well as empty corners around the PM. This tabular representation was also used with CNNs in Spier et al.^38^.

Three candidate MPI signals were considered for MACE prediction based on Dietz et al.^13^:

- sMBF: MBF values measured under stress $S_{\text {sMBF}}$ in ml/min/g,
- MFR values, obtained by taking the ratio between MBF values under stress and at rest for every element of the MBF matrix; that is $S_{\textrm{MFR}} [n,m] = \frac{S_{\text {sMBF}}[n,m]}{S_{\text {rMBF}}[n,m]}$ and
- MFC radius signal defined as $S_{\textrm{MFC}} [n,m] = \sqrt{S_{\text {sMBF}}[n,m]^2 + S_{\textrm{MFR}}[n,m]^2 }$ based on the initial findings of Dietz et al.^53^.

The first two signals (sMBF and MFR) are established methods for the quantitative assessment of myocardial perfusion and known to be predictive for MACE^13^. We introduce MFC radius as a simple geometric method to evaluate MFC at a pixel level to generate a “capacity” image^53^. We recall that MFC is able to leverage information from both sMBF and MFR^17^ and was previously reported to be predictive of MACE^13^. In what follows, we denote by *S* the measurement matrix, which is one of the three MPI signals introduced above. We also denote by $\varvec{y} \in \mathbb {R}^{n}$ the feature vector, where *n* is the number of considered features for the corresponding model. The various feature vectors $\varvec{y}$ considered are systematically detailed in the models’ descriptions below (i.e. segment-based or radiomics).

### Segment-based models

The first two models that we evaluated were based on two distinct parcellations of the considered PMs, hence of the $16 \times 36$ MBF matrices *S* (see Fig. 1). The global model of the PM corresponds to a global average over the matrix (i.e. over the entire LV), leading to the single-valued measure $y = \frac{1}{16 \times 36} \sum _{1 \le m \le 36, \ 1 \le n \le 16} S[m,n]$. We then learn a straightforward LR model taking the global average value of the matrix as unique feature. The 1-segment model was already reported to be predictive for MACE by Dietz et al.^13^ and will serve as our baseline. The second model is based on the standard 17-segment AHA model^16^ and corresponds to regional averages, yielding a vector $\varvec{y} \in \mathbb {R}^{17}$ of 17 features over each delimited region as in Fig. 1. All 17 regional features were standardized based on their respective averages and standard deviations of training samples.

### Radiomics models

Two radiomics models were considered. For the first radiomics model (referred to as “radiomics all”), a standard set of radiomics features was included^54^, excluding shape features as not relevant for analysing MPI. All features were computed over the entire measurement matrix *S* built based on one of the three parametric maps listed above. Using regional masks (e.g. 17 segments) could potentially limit the risk of aggregating radiomics features over too large regions and therefore discarding important local information^55^, but were not considered to limit the number of radiomics features and risk of overfit. We used intensity (18 features) as well as texture features including Gray Level Cooccurence Matrix (GLCM, 24 features), Gray Level Run Length Matrix (GLRLM, 16 features), Gray Level Size Zone Matrix (GLSZM, 16 features), Neighbouring Gray Tone Difference Matrix (NGTDM, 5 features), and Gray Level Dependence Matrix (GLDM, 14 features), for a total of 93 features yielding a vector $\varvec{y}\in \mathbb {R}^{93}$. The pyradiomics library was used^54^ with default settings and IBSI compatible in terms of feature definition^56^. We set the *binWidth* separately for each of the MPI signal considered (i.e. sMBF, MFR and MFC) as the max range of the values across the whole data divided by 16. All features were standardized based on their respective averages and standard deviations of training samples. The second radiomics model (referred to as “radiomics intensity”) was only based on the 18 intensity features and all texture features were left out, yielding a vector $\varvec{y}\in \mathbb {R}^{18}$. The two abovementioned radiomics models were used to investigate the specific value of spatial pattern analysis via texture features when compared to simpler intensity measurements.

### Machine learning

For all handcrafted models, i.e. global, 17-segment, radiomics all and radiomics intensity models, the respective feature vectors $\varvec{y}$ fed into either LR models for classification tasks or Cox Proportional Hazard (PH)^57^ regression models for predicting risks that are concordant with time-to-events. For either the LR or the Cox PH models, an ElasticNet penalty combining $L_1$ and $L_2$ regularization^58^ was optimized by 5-fold cross-validated grid-search over a range of $L_1 \_ \text {ratio}\in [0.50, 0.85, 0.9, 0.92, 0.95, 0.97, 0.98, 0.99, 0.995, 0.999, 1.0]$. This range was chosen to promote $L_1$ penalty and thus the sparsity of the model. The optimized score was the AUC or the Concordance (C)-index^59^ to determine the best $L_1 \_ \text {ratio}$ over the training set. For classification models, we represent the outcome *no MACE* (no event) versus *MACE* (event) by the output variable $z \in \{0,1\}$, so that we learn the parameters of the different LR on the $\left( \varvec{y},z\right)$ relations. For Cox PH models, time-to-event, events and censoring outcome data are provided the learn the prediction of hazard scores *z*. The LR and Cox PH models were chosen for their simplicity and well-established methods for radiomics studies. All computational operations were performed with Python v3.9.13, and mainly using scikit-learn v1.0.2 and TensorFlow v2.9.1 libraries.

#### Late fusion with clinical features

In order to investigate the predictive performance when combining clinical and imaging information, a late fusion of each distinct image model with a LR model based on 19 clinical features (see Material A), i.e. $\varvec{y} \in \mathbb {R}^{19}$ was implemented. To do so, the decision function was simply based on an unweighted average of the prediction scores (probabilities) of the considered image and clinical models. The clinical LR model was trained on the exact same splits as the image-based models (see Fig. 3). Categorical variables were transformed using one-hot encoding and all 19 clinical features were standardized based on their respective averages and standard deviations of training samples.

### AI models based on Convolutional Neural Networks

**Figure 2:**
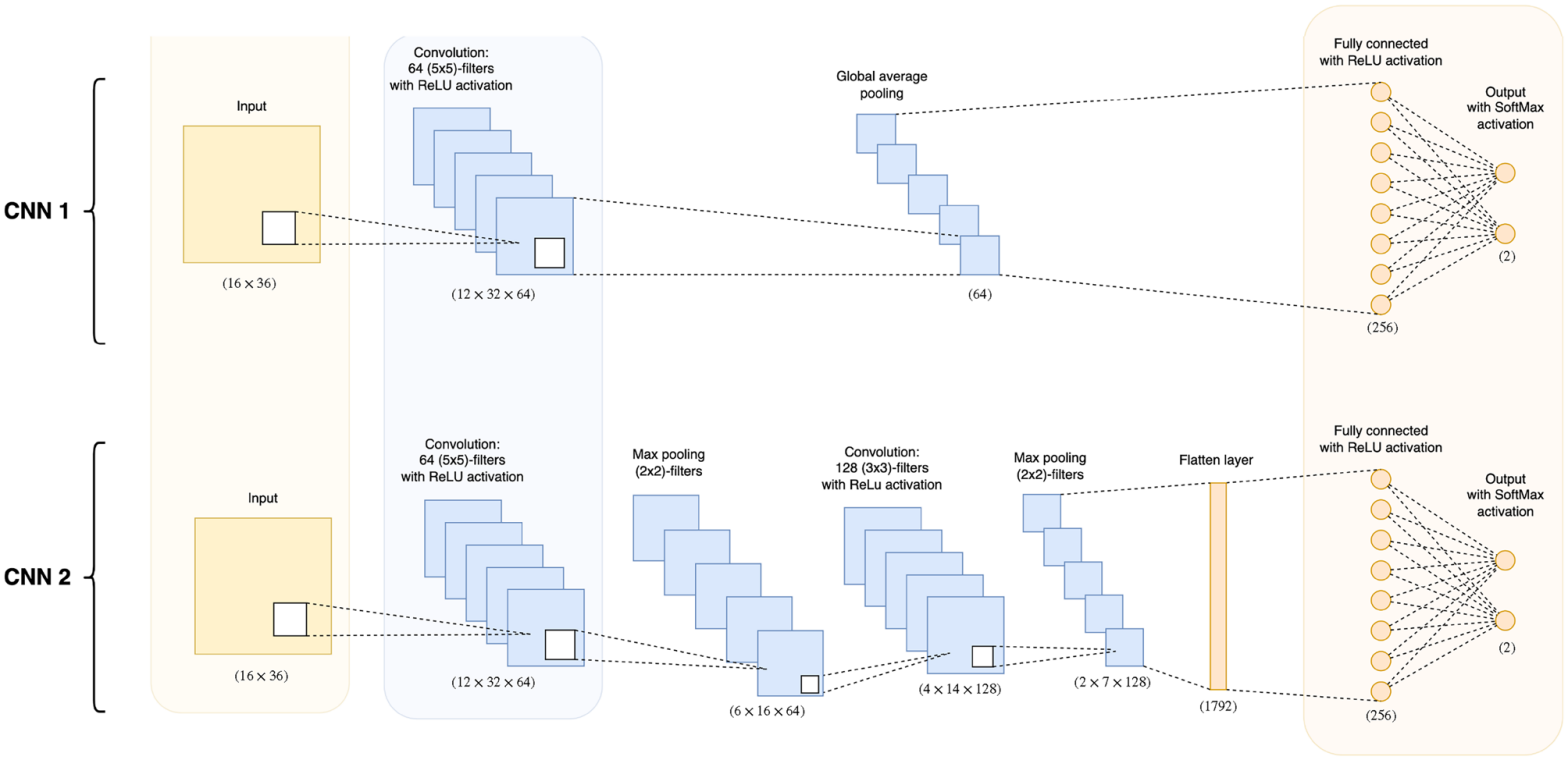
CNN architectures. CNN 2 was proposed by Spier et al. for MPI analysis based on the MBF matrix *S*^38^. The number of trainable parameters for CNN 1 and CNN 2 are 19k and 535k, respectively.

We explored the ability of CNNs to learn and capture more complex spatial patterns (see Fig. 2) as compared to the LR-based models. The measurement matrices *S* were used as input. We compared two shallow architectures with increasing depths (CNN 1 versus CNN 2). Deeper and pre-trained CNNs (i.e. Resnet50) have been subject to a preliminary evaluation which has not led to any improvement in results. Resnet50 is too complex (i.e. too many layers and parameters) with too large receptive fields to analyse the relatively small input images ($16\times 36$) considered in this work.

For all CNN models, an extra step consisting of data augmentation was included in the general pipeline, which was carried out after each train-test split. Since the acquisition of the images is very controlled, we only introduced minor transformations on the PMs simulating the effect of annotation uncertainty. In order to do so, the size of the training set was quadrupled using two types of transformations on the measurement matrices *S*, corresponding to small variations in the segmentation of the LV. The first transformation was to apply simple shifts of the columns of the matrix (on the right and on the left), corresponding to rotations of the PM of $\pm 10$ degrees (in both directions). Formally, we define the new matrix with the shift on the right by $S'$ with $S'_{ij} = S_{i(j-1)}$ for $j = 2,...,36$ and $S'_{i1} = S_{i36}$. Similarly, we define the new matrix with the shift on the left by $S''$ with $S''_{ij} = S_{i(j+1)}$ for $j = 1,...,35$ and $S''_{i36} = S_{i1}$. For the second transformation, the last 3 rows of the matrix (corresponding to the outer part of the PM) were erased, and the remaining 13 rows were stretched back to 16 rows using linear interpolation. This simulates variations in the adjustment of the segmentation of the LV on the PET-scan.

For the simplest CNN, referred to as “CNN 1”, we input the matrices and used a convolution layer with 64 filters of size $5 \times 5$, before the Global Average Pooling (GAP) layer. The architecture ended with a Fully Connected (FC) layer including 256 hidden neurons and 2 output neurons. The Rectified Linear Unit (ReLU) activation function was used for the convolutions and FC layers. A softmax activation function was used for the FC output layer (see CNN 1 in Fig. 2). For training the CNNs, we used a $L_1$-$L_2$ regularizer, an Adam optimizer, and a categorical cross-entropy as the loss function. 50 epochs were used for training.

A slightly deeper architecture was considered as used by Spier et al. to analyze SPECT-based MPI in^38^ (see “CNN 2” in Fig. 2). Similarly to the CNN 1 model, the MBF matrices were used as input followed by two successive convolution layers. The first one included 64 filters of size $5 \times 5$, and the second one had 128 filters of size $3 \times 3$. Each of them used a ReLU activation function, and was followed by a $(2 \times 2)$-max-pooling layer. A flattening layer was used before the FC layer including 256 hidden neurons and 2 output neurons. ReLU and softmax activation functions were used similarly to the previous model.

### General pipeline for AI model validation

**Figure 3:**
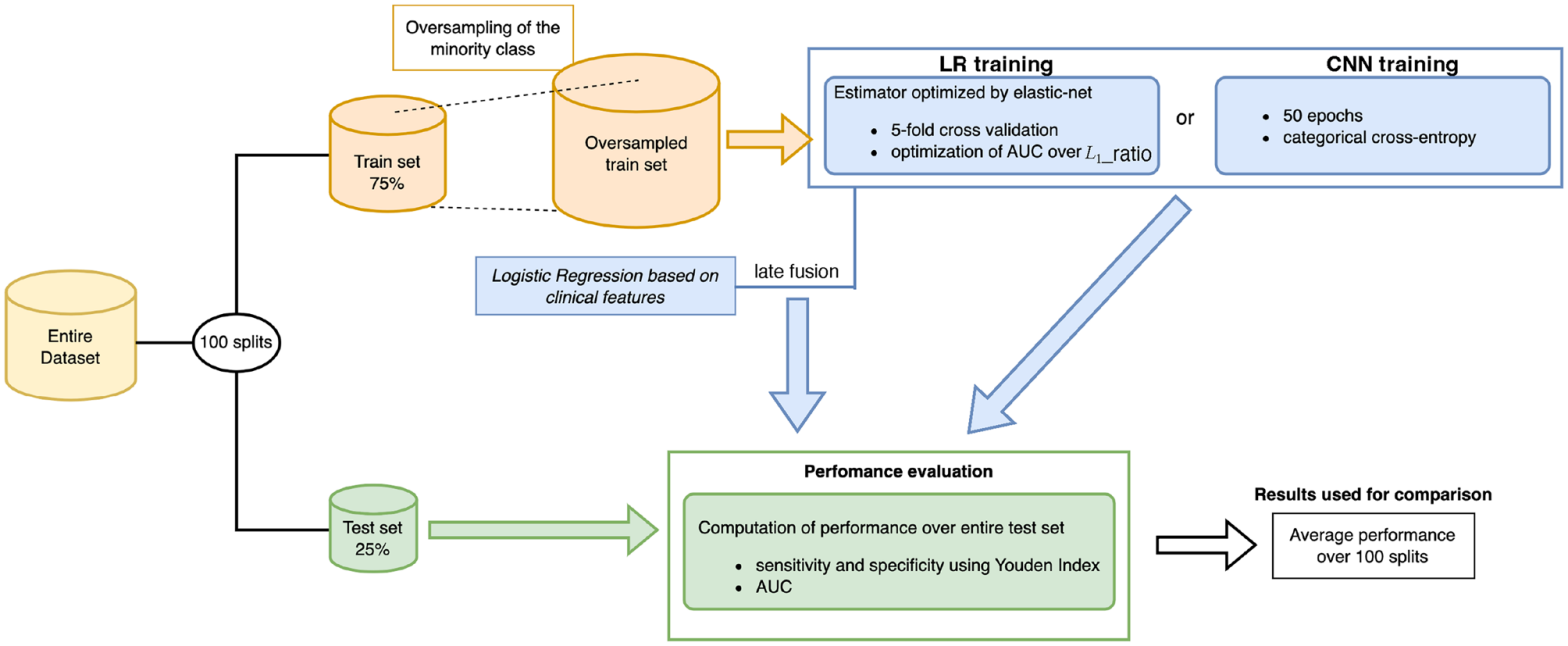
General pipeline for AI model validation.

In order to systematically compare the different models and assess performance variability, we followed the same procedure for each model family. We repeated 100 random stratified splits of the data into a training and a test set, with a 75–25% proportion (i.e. 175 training cases, including 35 positive, i.e. events, and 140 negative ones). Seeds were kept to ensure the use of same splits for all models. Within each split, the minority class was oversampled in the training set by triplicating every positive case, i.e. events, increasing the training size up to 245 cases (counting 105 positive and 140 negative cases). This was done to address the imbalance of our data, whose positive outcomes only represent a fifth of the total sample. For each tested model, the splits and the shuffling of the training set were exactly the same. The LR, Cox PH and CNN models were then trained, and their performances measured and stored for each split. For LR and CNN models, the AUC of the considered model was computed for each split, and the Youden index was used to determine the optimal cutoff value providing the optimal trade-off between sensitivity and specificity. We finally report the test average performances measured across the 100 splits. Confidence Intervals (CI) and tests for the average were built from 1000 bootstraps^60^ to compare all approaches. An overview of the general training and validation pipeline is depicted in Fig. 3.

## Results

**Figure 4:**
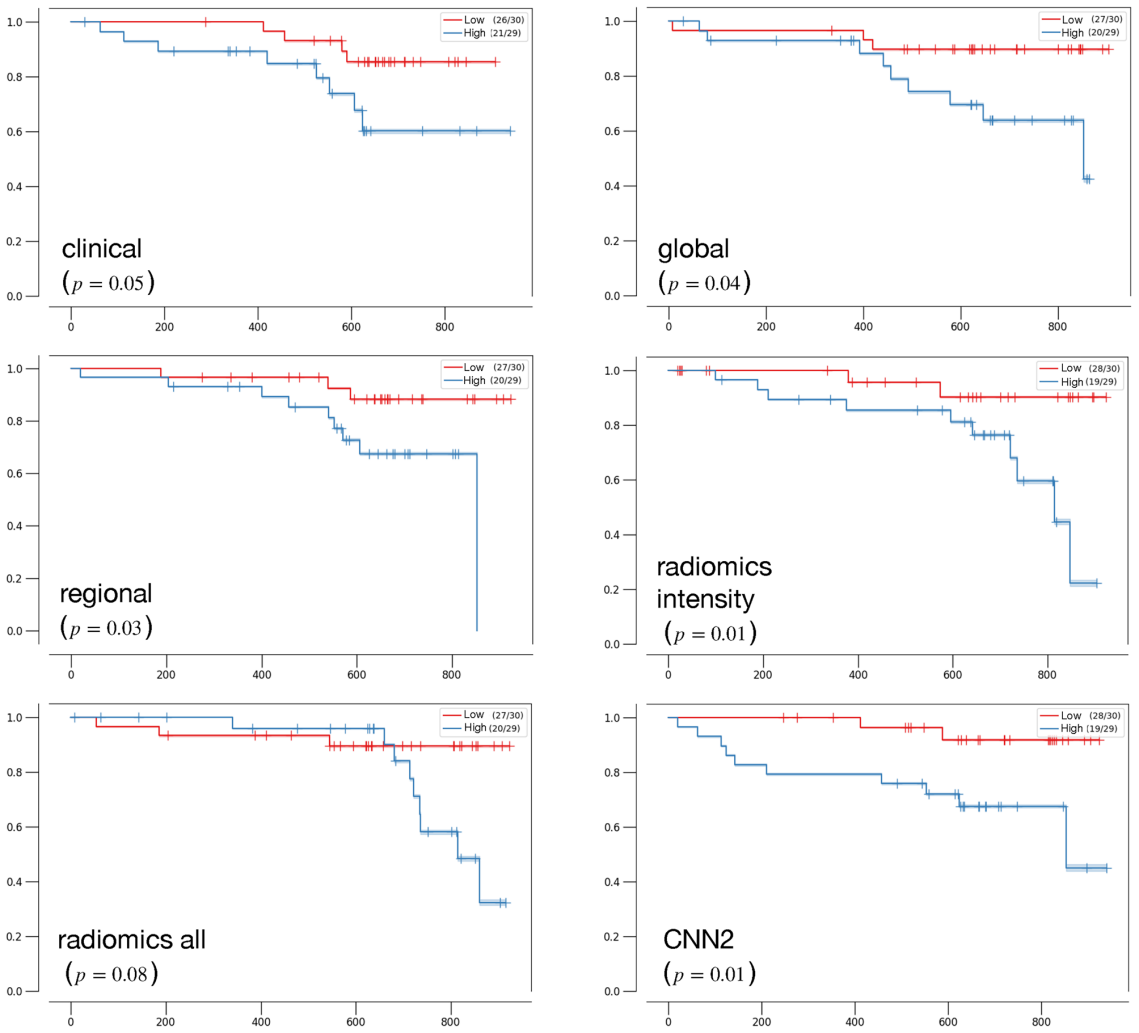
Kaplan–Meier analysis of the models. Low versus high risk groups are split based on the median score. The number of patients per group and censoring are indicated in brackets.

The demographics and MBF values of the included patients are detailed in Tables 1 and 2 of Dietz et al.^13^, respectively. Tables 3 and 4 summarize all the results for classification and Cox PH models, respectively when using sMBF, MFR and MFC radius. The LR model based on the 19 clinical features only achieves an AUC of 63.0% [61.5, 64.3], with a sensitivity of 70.6% and a specificity of 62.4%. The Cox PH model based on the clinical features only achieves a C-index 0.61 [0.60, 0.63]. Figure 4 compares Kaplan–Meier curves and log-rank tests based on the probability output scores of the clinical, global and CNN 2 classification models. The low versus high risk groups are based on the median of the probability output scores for each model.

**Figure 5:**
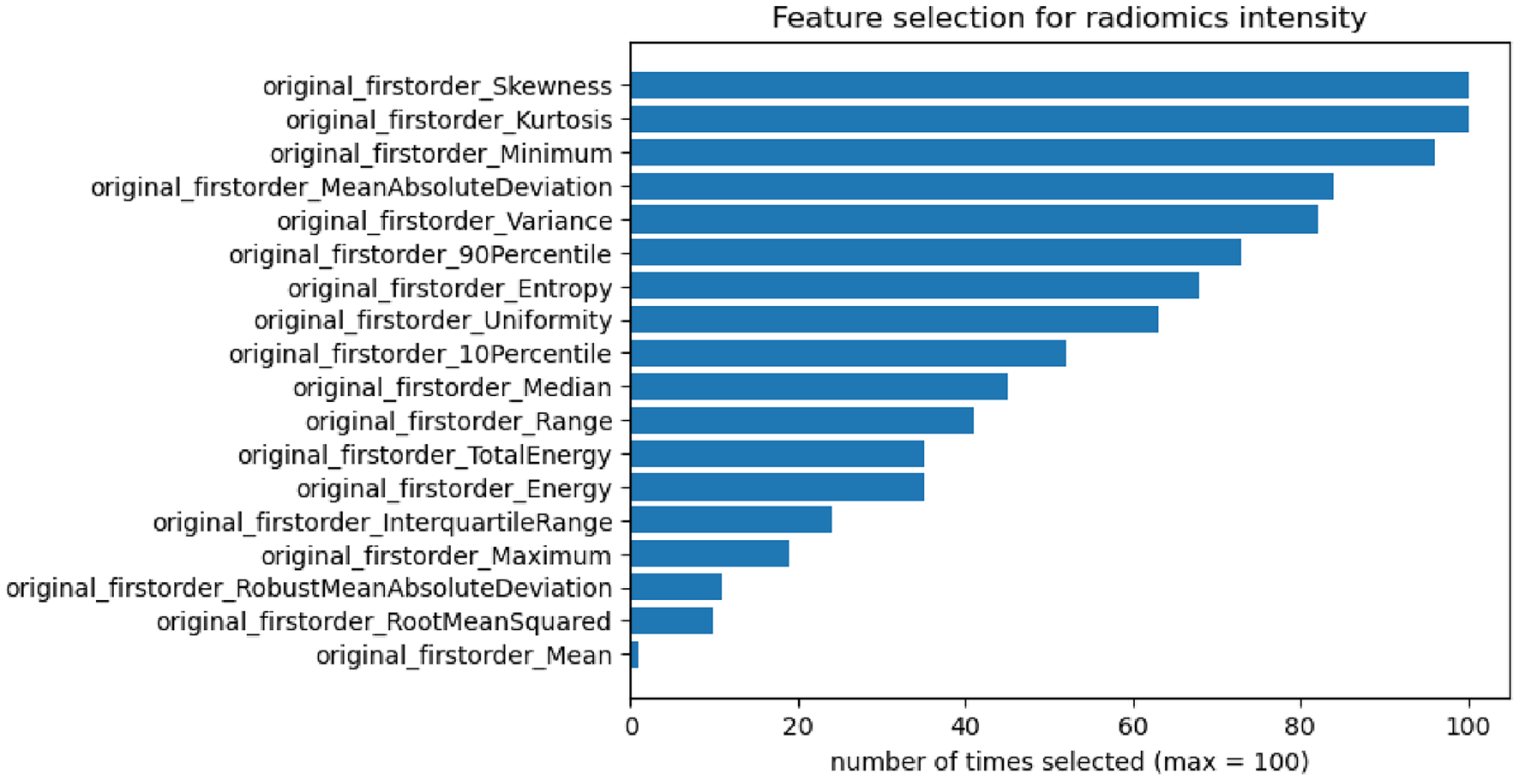
Features selection count for the MFC radius radiomics intensity model over the 100 repeated splits (see Fig. 3). Maximum number of times a feature can be selected is 100, i.e. at each split. The global average of the PM, referred to as “original_firstorder_Mean” is the one chosen least often.

Figure 5, reports the set of features selected by ElasticNet for the radiomics intensity model across all 100 split repetitions (see Fig. 3).

**Table 3:** Average test performance (in %) of the models computed across 100 stratified random splits.

Model	# feat.	sMBF			MFR			MFC radius		
		AUC	Sens.	Spec.	AUC	Sens.	Spec.	AUC	Sens.	Spec.
Global	1	68.3 [67.0, 69.7]	80.4	62.4	69.7 [68.4, 71.2]	72.5	69.4	70.5 [69.4, 71.8]	77.4	66.0
Regional	17	71.2 [69.6, 72.4]	78.1	65.3	71.6 [70.4, 72.9]	74.3	69.8	73.4 [72.3, 74.7]	74.8	**72.0**
Radiomics all	93	67.4 [66.0, 69.0]	72.3	64.7	68.7 [67.2, 70.4]	73.1	67.5	71.6 [70.0, 73.0]	72.4	71.1
Radiomics intens.	18	**73.2** [71.6, 74.5]	77.1	**70.2**	68.4 [66.9, 69.8]	73.3	66.8	73.8 [72.4, 75.1]	76.4	67.7
CNN 1	–	70.0 [68.5, 71.3]	80.1	64.3	70.3 [69.0, 71.8]	74.4	**71.1**	73.6 [72.4, 74.8]	74.8	71.0
CNN 2	–	71.3 [69.7, 72.8]	**81.4**	64.9	70.8 [69.4, 72.2]	77.3	66.4	**73.9** [72.5, 75.3]	77.5	69.0
Clinical + global	20	67.6 [66.3, 68.9]	77.6	61.6	68.5 [67.2, 69.8]	75.2	64.6	69.1 [68.0, 70.3]	79.0	62.8
Clinical + regional	36	70.9 [69.4, 72.1]	78.2	65.3	**71.8** [70.6, 73.0]	76.9	68.4	72.5 [71.3, 73.8]	76.5	70.0
Clin. + rad. all	112	69.1 [67.7, 70.5]	72.9	67.1	70.8 [69.1, 72.3]	78.0	67.1	72.8 [71.2, 74.1]	**80.0**	67.4
Clin. + rad. intens.	37	71.5 [70.2, 72.8]	76.7	65.9	69.9 [68.5, 71.3]	77.9	64.5	72.7 [71.3, 73.8]	**80.0**	67.7
Clin. + CNN 1	–	68.2 [66.8, 69.4]	77.7	62.4	69.3 [68.0, 70.5]	76.7	64.7	70.0 [68.9, 71.3]	78.5	64.3
Clin. + CNN 2	–	70.4 [69.2, 71.6]	80.1	63.1	71.2 [70.0, 72.5]	**78.5**	66.4	72.1 [70.9, 73.3]	78.5	67.7

Bootstrap-based 95% confidence intervals are reported for AUC. A LR model based on the 19 clinical features only achieves an AUC of 63.0% [61.5, 64.3], with a sensitivity of 70.6% and a specificity of 62.4%. The top performance is highlighted in bold for each column.

**Table 4:** Average test C-indices of the Cox PH models computed across 100 stratified random splits.

Model	# feat.	sMBF	MFR	MFC radius
Global	1	0.65 [0.64, 0.66]	0.68 [0.66, 0.69]	0.68 [0.67, 0.69]
Regional	17	0.66 [0.65, 0.68]	0.69 [0.68, 0.71]	0.71 [0.70, 0.72]
Radiomics all	93	0.66 [0.65, 0.68]	0.66 [0.64, 0.67]	0.69 [0.68, 0.71]
Radiomics intens.	18	0.67 [0.66,0.69]	0.67 [0.65,0.68]	**0.72** [0.71, 0.73]
Cinical + global	20	0.64 [0.63, 0.66]	0.67 [0.65, 0.68]	0.66 [0.64, 0.67]
Clinical + regional	36	**0.68** [0.67, 0.69]	**0.70** [0.68, 0.71]	0.71 [0.70, 0.72]
Clin. + rad. all	112	0.66 [0.65, 0.68]	0.67 [0.66, 0.68]	0.69 [0.68, 0.71]
Clin. + rad. intens.	37	0.66 [0.65, 0.67]	0.67 [0.66, 0.68]	0.69 [0.68, 0.71]

Bootstrap-based 95% confidence intervals are reported. A Cox PH model based on the clinical features only achieves a C-index 0.61 [0.60, 0.63]. The top performance is highlighted in bold for each column.

## Discussions

In this study, we investigated the value of AI for analyzing [^82^Rb] PET-based MPI in order to predict MACE. To this end, we systematically implemented and compared standard AI approaches with a global validation pipeline. In particular, we used LRs and Cox PH models based either on MBF segmental (global or regional based on 17 segment) or radiomics (with and without texture) features, as well as shallow CNNs. We also considered a late fusion of imaging signature (global, local, radiomics or CNN) with a LR model uniquely based on clinical features. The three considered input MPI signals including sMBF, MFR and MFC radius were also compared for all models. The training and the evaluation of all these approaches was carried out with a systematic validation pipeline allowing fair and reproducible comparisons between the approaches. The respective performance of models and MPI signals were evaluated based on AUC, sensitivity, specificity and C-index estimated across 100 random train/test splits of the original data.

We interpret the comprehensive performance comparisons presented in Tables 3 and 4 as follows. First, the use of sMBF or MFR seems to perform equivalently on most of the models, whereas for the MFC radius, the performance is consistently better. When focusing on the models based on MFC radius, we observe a significant improvement in average performance when using the regional information across the 17 segments versus using the global average alone (average AUCs of 73.4% and 70.5%, respectively, $p<0.001$ and average C-indices of 0.68 and 0.71, respectively, $p<0.001$). Next, we note that the models including clinical features do not yield higher AUCs or C-indices than models that purely rely on image information. For MFC radius, the late fusion seem to consistently improve sensitivity, at the high cost of decreased specificity and resulting in overall decreased AUC. Thus the added value to combine clinical information with image models remains to be confirmed where more advanced approaches to fuse the two sources of information could be explored. Finally, we observe that the models including texture information such as CNNs and radiomics all do not systematically outperform models based on simpler intensity features (i.e. segment-based and radiomics intensity models). For instance, the performance of the 17-segment regional model (average AUC of 73.4%) was found not to be significantly inferior ($p=0.33$) to the one of the CNN2 model (average AUC of 73.9%). It indicates that the texture information (i.e. subtle spatial patterns that are mostly invisible to the naked eye) contained in the MBF matrices may not predict MACE better than intensity information alone, and that the ElasticNet models do not fully succeed to select most relevant features in the case of the radiomics all model. This suggests that the spatial characteristics of MBF patterns may not be relevant for MACE prediction. It is worth noting that the texture features were aggregated over the entire MBF matrix, and (3- or 17-) segmental aggregation was not investigated to limit the numbers of features per patient. When analyzing intensity features that are retained in the radiomics intensity model in Fig. 5, it is remarkable that the mean feature from the first-order statistics, being strictly equivalent to the global model, is the feature that is the least often selected. This suggests that the spatial aggregation of the MBF signal requires more sophisticated strategies than global averaging. Skewness, kurtosis and the minimum of the MBF distribution constituted the group of most predictive intensity features. From a physiopathological perspective, the superiority of intensity features over texture features is expected since [^82^Rb] PET-based MPI provides an absolute quantitative assessment of perfusion capacity and deficits that are related to MACE risk. The Kaplan–Meier analyses reported in Fig. 4 demonstrate that all AI models but radiomics all result in better separability of low versus high risk groups when compared to the global and clinical models.

## Conclusions

Our study demonstrates for the first time the feasibility of AI-based approaches using dynamic [^82^Rb] PET/CT data in the assessment of MACE, using quantitative MBF parameters as gold standard. Radiomics intensity and CNN 2 models achieved promising results with observed average AUCs of 73.8% and 73.9%, respectively. Overall we conclude that the regional model constitutes an excellent trade-off between model simplicity and performance, achieving high AUC (73.4%) and best specificity (72%). In comparison, CNN 2 (best AUC of 73.9%) favors sensitivity over specificity. The results are promising, and the moderate diagnostic accuracy achieved by the models could be explained by the following limitations.

This study is based on a single center with a relatively limited sample size. It would be of interest to apply this pipeline to a larger database collected across multiple centers. We used normalizing resting flow according to the pressure-rate product, which could inherently reduce the prognostic value of flow reserve to predict MACE risk. While this first study focused on traditional AI methods (i.e. radiomics with LR and simple NNs), more advanced AI methods will be investigated in future work. In particular, the tabular representation (i.e. MFB matrix) used as input to the models suffers from spatial distortion. It is worth noting that using PMs as input would also involve spatial distortions in the apex region. Nevertheless, future work will consider analyzing the 3D PET images or graph-based CNNs as this approach showed to better leverage the spatially distorted content of PMs for MPI abnormality classification in SPECT-based MPI^38^. Other quantitative measurement from [^82^Rb] PET can also be considered such as left ventricular ejection fraction^61^. Another limitation of this study lies in the imbalance of the data, even though we reduce its impact by addressing the lack of positive cases with the oversampling of the minority class.

### Supplementary Information

Supplementary Information.

## Data Availability

The datasets generated and/or analysed during the current study are not publicly available as not permitted by the ethics agreement.
