# Supplementary material for: Comparing various AI approaches to traditional quantitative assessment of the myocardial perfusion in [82Rb] PET for MACE prediction
Source: Sci Rep. 2024 Apr 26;14:9644. doi: 10.1038/s41598-024-60095-6 (PMC11053111; doi:10.1038/s41598-024-60095-6)
Supplement: Supplementary file 1 — Supplementary Information. [file 41598_2024_60095_MOESM1_ESM.pdf]

A Supplementary Material: List of clinical features

1. Age
2. Sex
3. Weight
4. Size
5. Body mass index (Kg/m2)
6. Hypertension
7. Current or former smoker
8. Dyslipidemia
9. Diabetes
10. Insulin-dependant diabetes
11. Family history of early CAD
12. Known CAD
13. History of myocardial infarction
14. Aspirin
15. Beta-blockers
16. Angiotensin-converting enzyme inhibitors
17. Diuretic
18. Nitroglycerine therapy
19. Lipid-lowering agent
